# Exploring health and well-being in Taiwan: what we can learn from individuals’ narratives

**DOI:** 10.1186/s12889-020-8201-3

**Published:** 2020-02-03

**Authors:** Patricia Rodriguez Espinosa, Yong-Chen Chen, Chien-An Sun, San-Lin You, Jaw-Town Lin, Kun-Hu Chen, Ann W. Hsing, Catherine A. Heaney

**Affiliations:** 1 Stanford Prevention Research Center, Stanford University School of Medicine, 3300 Hillview Ave (Mail code: 5537), Palo Alto, CA 94304 USA; 2 School of Medicine, College of Medicine, Fu-Jen Catholic University, No. 510, Zhongzheng Rd., Xinzhuang District, New Taipei City, Taiwan; 3 Big Data Research Center, College of Medicine, Fu-Jen Catholic University, No. 510, Zhongzheng Rd., Xinzhuang District, New Taipei City, Taiwan; 4 Department of Public Health, College of Medicine, Fu-Jen Catholic University, No. 510, Zhongzheng Rd., Xinzhuang District, New Taipei City, Taiwan; 5 Department of Clinical Psychology, Fu-Jen Catholic University, No. 510, Zhongzheng Rd., Xinzhuang District, New Taipei City, Taiwan; 6 Stanford Cancer Institute, Stanford University School of Medicine, 780 Welch Road, CJ Huang Building, Palo Alto, CA 94305 USA; 7 Department of Epidemiology, Stanford University, Palo Alto, CA USA; 8 Department of Psychology, Stanford University, 450 Serra Mall, Building 20, Palo Alto, CA 94305 USA

**Keywords:** (3–10 for indexing purposes): Well-being, Health, Qualitative inquiry, Health behaviors, Family, Taiwan

## Abstract

**Background:**

Our aim was to explore the concepts of health and well-being from the point of view of the people experiencing them. Most of the efforts to understand these concepts have focused on disease prevention and treatment. Less is known about how individuals achieve health and well-being, and their roles in the pursuit of a good life. We hoped to identify important components of these concepts that may provide new targets and messages to strengthen existing public health programs. An improved understanding of health and well-being - or what it means to be well - can guide interventions that help people lead healthier, more fulfilling lives.

**Methods:**

Using a grounded qualitative approach drawing from narrative inquiry, we interviewed 24 Taiwanese adults. Thematic inductive coding was employed to explore the nature of health and well-being.

**Results:**

Eight constituent domains emerged regarding well-being and health. While the same domains were found for both constructs, important frequency differences were found when participants discussed health versus well-being. Physical health and lifestyle behaviors emerged as key domains for health. Disease-related comments were the most frequently mentioned sub-category within the physical health domain, along with health care use and aging-related changes. For well-being, family and finances emerged as key domains. Family appears to be a cornerstone element of well-being in this sample, with participants often describing their personal well-being as closely tied to - and often indistinguishable from - their family. Other domains included work-life, sense of self, resilience, and religion/spirituality.

**Conclusions:**

Health and well-being are complex and multifaceted constructs, with participants discussing their constituent domains in a very interconnected manner. Programs and policies intended to promote health and well-being may benefit from considering these domains as culturally-appropriate leverage points to bring about change. Additionally, while the domains identified in this study are person-centered (i.e., reflecting the personal experiences of participants), the stories that participants offered provided insights into how well-being and health are influenced by structural, societal and cultural factors. Our findings also offer an opportunity for future refinement and rethinking of existing measurement tools surrounding these constructs.

## Background

Over 70 years ago, the World Health Organization (WHO) defined health as “a state of complete physical, mental, and social well-being, and not merely the absence of disease or infirmity” [1]. However, most of the efforts of health care providers and public health professionals have continued to focus on disease prevention and treatment. While these efforts have contributed to an increase in life expectancy and improved disease management [2], they overlook much of the WHO definition. If health is more than the absence of disease or infirmity, what exactly is it and how do individuals achieve it? And what role does health and well-being, however defined, play in the pursuit of a fulfilling life?

To address these questions, some scholars have turned to the concepts of quality of life [3] or health-related quality of life [4]. Others have used the phrase “health and well-being” to denote a broad, inclusive approach to studying and promoting a life well-lived. Too often the terms or phrases that are used are not clearly defined and when they are, it is often through the lens of a particular academic discipline. For example, economists tend to emphasize objective indicators of economic function and disease [5]. Psychologists tend to focus on subjective experience, emotions and cognitions [6, 7]. Medical professionals have focused on the experience of symptoms and functional capacity, both physical and mental [8]. Health promotion professionals have concentrated on lifestyle choices and behaviors such as sleep, nutrition, and physical activity [9].

In this study, we aim to dispense with academic disciplinary lenses and instead use a grounded inquiry approach in order to explore the concepts of health and well-being from the point of view of the individuals experiencing them. Such an approach assumes that these concepts are inherently subjective (i.e., one cannot experience good health or high well-being without perceiving it as such)*.* However, it assumes little else. This approach allows individuals to think beyond diseases and constraints of the health care system and to incorporate everyday life experiences, allowing for the emergence of themes that might be critically important to individuals’ well-being but are not currently captured by more discipline-specific, expert-driven efforts. Our approach also encourages individuals to reflect on both positive and negative aspects of experiences that may contribute to or detract from health and well-being. This is in concert with the WHO definition of health and avoids the sole focus on deficits and problems that permeates many of the prior efforts to conceptualize health and well-being [4].

Another important benefit of using a grounded approach is that it is open to the contributions of people from different cultures and with greatly varying life experiences. Much of the existing well-being literature relies on measures primarily validated with data from western nations [10, 11], while the small number of studies in Asian countries have tended to be in Japan [12] or China [13]. Given that the meaning of health and well-being, or a “good life,” can vary across countries and cultures, the use of translated measures, without proper validation from the local culture and context, may result in misleading findings being used to inform important policy decisions [14].

An improved (and perhaps more comprehensive) understanding of health and well-being - or what it means to be well - can guide interventions that aim to help people lead healthier, more fulfilling lives. By identifying key themes or domains of these concepts and potential interconnectedness among them, we may be able to adapt and strengthen existing public health policies and programs. For example, existing efforts have been relatively unsuccessful in bringing about sustainable health behavior changes that can curtail current public health problems associated with obesity, physical inactivity, and other chronic disease risk factors [15, 16]. A better understanding of what individuals find motivating for living healthy fulfilling lives may provide new targets and messages for change. By “starting where the people are” [17], we may enhance the effectiveness of public health and health promotion efforts.

The Wellness Living Laboratory (WELL) at Stanford University has launched a global research effort, known as WELL for Life, with the mission to accelerate the science of well-being and optimize health and well-being for all. This global initiative has partnerships in China, Singapore, Taiwan, and Thailand. Asian countries represent nearly 60% of the world population and suffer from high burdens of disease [18, 19], making any efforts in the region potentially hugely impactful. Moreover, the United Nations has made ensuring health and well-being in this region a sustainable development goal [20], making this study timely and relevant.

Taiwan, a prominent East Asian country, has seen multiple societal changes since the 1960s, including urbanization, industrialization, lower fertility rates, and increasing life expectancy [21–23]. As a traditional East Asian society, collectivistic values are likely to be prevalent [24, 25], including an emphasis on social relationships, group harmony, collective identity, and filial piety [26]. A small literature has explored how such values may influence notions of health and well-being but few of these studies have been conducted in Taiwan (for example [22, 23]). As the cross-cultural study of health and well-being continues to burgeon, studies conducted in countries not previously well represented in this specific literature can make key contributions to identifying broad commonalities, as well as points of divergence, among the nations of the world.

### Objectives

The present study aims to better understand how Taiwanese adults discuss the nature of the concepts of health and well-being. By concentrating on stories from individuals’ lives, using a grounded approach that draws on narrative inquiry methods, we aim to generate new knowledge about the important components of these concepts and what it means to “be well.”

## Methods

Qualitative methods are well-suited for research questions surrounding the meaning of personal experiences and for uncovering differences in conceptualizations due to cultural or contextual factors [27]. This study borrows from narrative inquiry [28]. Increasing in popularity for use in social research, this person-centered approach uses participants’ personal stories or narratives to understand and give representation to phenomena – events, experiences, thoughts, feelings. These stories can offer insights into social and cultural meanings and patterns [29].

### Sample and settings

As part of the WELL for Life initiative, in collaboration with Fu-Jen Catholic University (FJU) in Taiwan, participants were recruited in New Taipei City using a snowball and convenience sampling approach [30, 31]. Inclusion criteria included: 1) adult residents of New Taipei City, Taiwan, aged 30–79, 2) ability to speak Mandarin, and 3) willingness to share their personal stories as related to health and well-being. Efforts were made to maximize variation in terms of gender, age and socio-economic status. The total sample consisted of 24 adults (54% female) ranging in ages from 31 to 66. While there is no established consensus on sufficient sample size for qualitative studies, our sample is consistent with those in the qualitative interview-based literature. A recent review of sample sizes in qualitative health research found median sample sizes between 15 and 31 across several journals [32]. Study designs, such as ours, that employ open-ended questions are likely to produce rich contextual data for each participant, requiring a smaller overall sample size [32]. Additionally, saturation (i.e., no new information emerging from additional interviews) was employed to guide the need, or lack thereof, for additional cases [32].

Full demographic characteristics are shown in Table 1. Most of the participants were either employed (75%) or self-employed (21%), and almost all participants lived with family members (92%). Data from New Taipei City Statistics Database [33] indicates that our sample is generally reflective of the variability one would typically find in New Taipei City in terms of gender distribution, marriage rates, and educational attainment.

**Table 1:** Participants’ Socio-Demographic Characteristics

	Total sample (*N* = 24)
Age in years (mean and range)	50.9 (31–66)
Gender (% female)	54%
Marital status (% married)	83%
Have children (% yes)	79%
• Average number of children	1.2 (range 1–4)
Education	
• High school or less	66.7%
• College	20.8%
• Master or above	12.5%
Living with family	92%
Employment status	
• Employed	75%
• Self-employed	21%
• Retired	4%
Religious affiliation	
• Chinese system of beliefs	54%
• Buddhism	17%
• Multiple affiliations ^a^	21%
• Non-religious	8%
Well-being ladder mean rating (range)	5.8 (1–8) ^b^
Health ladder mean rating (range)	5.2 (1–8) ^b^
Participants with same ladder ratings for well-being and health (%)	8 (33%)

^a^Multiple religious affiliations endorsed by participants include combinations of Chinese belief system, Buddhism, and Christian

^b^1 = lowest level to 8 = highest or best level for well-being and health, respectively

### Procedures

Individual semi-structured interviews were conducted at locations convenient to the participants. Following the interview, participants completed brief questionnaires regarding socio-demographic information. Participants received a gift card of $200 Taiwan Dollars ($6.5 USD) as compensation for their time. Informed consent was obtained from all individuals included in the study.

#### Translation process

Given the multifaceted nature of well-being, arriving at a proper translation of the term poses challenges. After careful consideration, 幸福 or 幸福感 (noun version) were chosen to represent “well-being.” These terms best translate to “happiness” or “content,” which we believe more closely encompasses well-being compared to alternatives (e.g., the WHO-5 Chinese version uses 身心健康, which translate as “physical and mental health”). We believe that using a translation term that cues participants to think about mental and/or physical health could potentially discourage discussion of other aspects of well-being. We used the term 整體健康 for health, which translates to “overall health” but can also be interpreted as overall physical and mental health. The FJU team and Taiwanese Stanford team members who speak Mandarin fluently guided and tested the translations.

#### Interview protocol

Using a grounded approach that draws from narrative inquiry, participants were asked to share stories regarding times of particularly high and low well-being. Similarly, later in the interview, they were asked to share stories regarding times of particularly good and poor health. The interviewer did not define the word “well-being” or “health,” but allowed participants to share what the words meant for them in the context of their lives via stories. If clarification was requested, the interviewer stated that the purpose of the study was to learn about participants’ perspectives and reiterated that there were no right or wrong answers. On average, interviews were 33 min in length. Participants discussed well-being in more depth than they did health, with the portions of the interview lasting an average of 23 min and 10 min respectively.

Additionally, using a ladder symbol with eight rungs, participants were asked to self-rate their current levels of well-being and health by choosing a rung on the ladder and to elaborate on the reasons for the chosen rating. This was adapted from Cantril’s Ladder of Life Satisfaction [34, 35], with higher rungs representing higher well-being or better health.

### Data analysis strategy

Interviews were audio recorded and transcribed verbatim. Using professional transcription and translation services, interviews were first transcribed in the original language and then translated into English for coding purposes. Transcriptions were checked multiple times and translations discussed before the coding process started. Translation from Chinese transcripts to English was conducted by a certified translator and quality controlled by a Stanford team member who is Taiwanese American and is fluent in both Mandarin and English. Taiwanese members of the research team were consulted for any issues related to the transcripts and subtleties of translating more complicated phrases (e.g., idioms and metaphors) used by participants.

#### Coding

We employed an inductive coding approach [27, 36], which allows findings to emerge from the raw data without imposing an a priori data structure. Three members of our research team read and coded transcripts and developed a codebook using an iterative approach. The codebook contained codes, definitions, examples, and the coding structure (e.g., overarching codes, sub-codes) to guide and ensure consistency among coders. Sections of text (called *data elements* from hereon in) could be assigned single codes or multiple codes if several ideas were articulated. Using thematic analysis [37], codes were later organized into themes or domains (some with sub-codes) for both well-being and health. Team meetings were used to discuss the coding approach; develop, revise and finalize our codebook; and for general training and clarification. Analytic memos were also employed to ensure rigor and to document the rationale for themes and data organization strategies. We used NVivo Mac version 12 software [38], for all data analysis and coding.

Formal inter-coder reliability was assessed using kappa coefficient measures [27]. The kappa value in this study was 0.92, which represents excellent agreement. In addition to this formal assessment of inter-coder reliability, during the initial coding process, coders met on a weekly basis and discussed their line by line coding for specific interviews. This was used as an opportunity to ensure that coders were using the codebook similarly and to resolve any questions and provide clarification on any codes or concepts. Instances of disagreement were discussed and resolved in team meetings. Post-coding analyses included exploration of the inter-relatedness among domains (i.e., co-occurrence of codes), the extent to which participants discussed each domain in terms of contributing to or detracting from their health and well-being, and the extent to which participants mentioned each domain.

## Results

Participants shared a variety of stories when discussing health and well-being. The large majority told stories related to major life events (e.g., getting married, the birth of a child, losing a loved one, purchasing a home), followed by stories surrounding a stage or time in their lives such as being a student, a certain time in their careers, or life after retirement. All participants spoke about other people and their roles in their lives, particularly family members. Many of the narratives also included routines or activities participants typically engage in, or used to engage in, such as hobbies, quality time activities with loved ones, and job-related activities. These stories also tended to highlight participants values, beliefs, identities and life goals. Next, we attend to ‘small story’ narratives [29] and draw out specific themes or domains related to health and well-being.

### Well-being and health domains

**Fig. 1:**
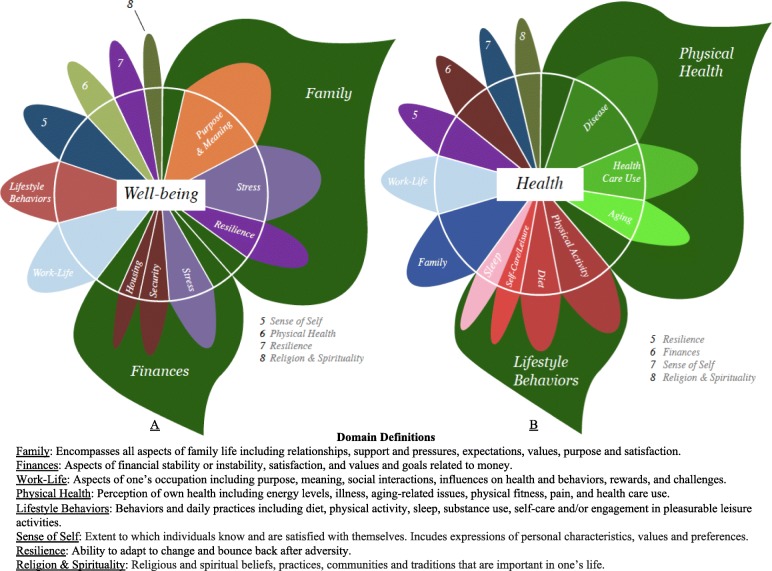
Domains of well-being and health and their definitions

**Fig. 2:**
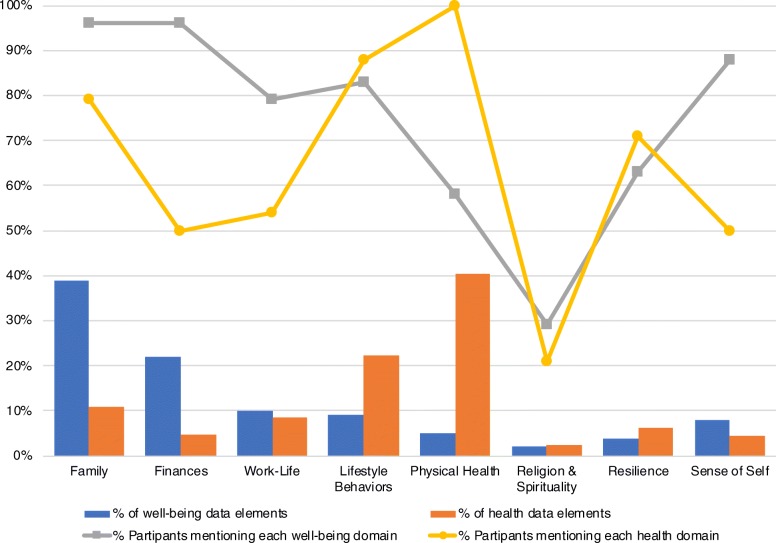
Percent mentions for well-being and health and percent of participants mentioning each domain *Legend*. Percentages were calculated separately for each portion of the interview. Well-being included a total of 883 data elements. The health portion of the interview included a total of 454 data elements

A total of eight domains emerged from our data regarding well-being and health. While the same domains emerged for both constructs, participants spoke about different domains with different levels of frequency during the two sections of the interview (e.g., well-being and health). Figure 1 showcases the domains separately for well-being and health, arranged by decreasing frequency of mentions for each domain. This figure also showcases definitions for each domain on the lower panel. Figure 2 highlights the percent of participants mentioning each domain, and the percentage of data elements coded under each domain. Notably, participants spent substantially more time discussing well-being (883 data elements) as compared to health (454 data elements). Calculations were done separately for well-being and health. Figure 2 displays percentages calculated based on these values. The following sections delineate findings separately for well-being and health.

#### Well-being findings

Figure 1, panel a, shows the well-being “flower” illustrating the domains of well-being. Larger petals correspond to higher numbers of data elements assigned to that code. Leaves were drawn to represent and draw attention to key domains most often discussed by participants. Petals within leaves represent sub-themes within the domain depicted by the leaf. Figure 1 also presents all domain definitions.

##### Family

For well-being, family emerged as the most frequently discussed domain (mentioned by 96% of participants when discussing well-being and accounting for 39% of all well-being data elements). Family appears to be a cornerstone element of well-being in this sample, with participants often describing their personal well-being as intimately tied to - and often indistinguishable from- their family. For instance, when discussing times of personal high and low well-being, participants mentioned the well-being of their children, the health of family members, their relationships with spouses and parents, family members as motivators to engage in health promoting activities, and other aspects of family life. The following quote highlights the intricate connection between family and participants’ well-being:

“*I am more of a family man. The old concept is always inseparable from home. After getting married, with children, we continue to live with my parents. The home gives me the feeling like sheltering from the wind. And this kind of warmth is my first choice of well-being*.” Male participant

Within the family theme, purpose and meaning was the largest sub-theme (see petal inside family leaf, Fig. 1, panel a). Participants mentioned family as the key driver in terms of finding purpose and meaning in their everyday lives. Stress was the second largest sub-theme within family, followed by resilience. Participants discussed stressors in the family including physical health problems of family members, conflicts with in-laws, stressors associated with raising young children and others. Nonetheless, families appear to provide invaluable support, as highlighted by the following quotes:

“*Like for my operation, I didn't take the sick leave for a month, but my husband did for a month to take care of me, very sweet. After the operation, he didn’t know how to cook, so he asked my sister to come over and help. He helped buying food, but he wouldn't cook. So, he would help prepare, and wait for my return for work to cook, but I don't have to do the dishes after dinner. He did them all. After operation, he would help me shower. He took care of me. He is very caring. So, I think I am very content*.” Female participant

“*Because the old house was not good at that time, we bought a house in the south and have everyone lived there with us. The in-laws and the uncles lived with us, and we were responsible for the payment of the house loan. Later we moved out. They still live there, even though we paid for it. But that loan was not from the bank. It was from a relative, no interest. It was much less pressure*.” Another female participant

Participants also discussed, with less frequency (and thus not explicitly depicted on Fig. 1), other family-related issues and their impact on well-being including changes in cultural values related to families (e.g., effects of nuclearization of families, changes in respect for elders), emotions evoked by one’s family (e.g., deep joy and happiness at seeing one’s children do well), and social comparisons related to being married or having children as desirable outcomes that were seen as important for attaining higher well-being.

##### Finances

The second most discussed well-being domain was finances. When speaking about their well-being, this domain was mentioned by 96% of participants and accounted for 22% of the data elements for well-being. Financial stress emerged as the largest sub-theme within finances. Our participants discussed various stressors such as the financial pressures of affording general life expenses; starting and failing in business; issues with employment, loans and debt; and others. The following quote highlights some of the impacts of financial issues:

A male participant described the following when discussing having failed in a business and incurring financial stress as a result:

“*I used to be in tears when I talked about this. It’s so pitiful. It went down just like this. And, it would take me another 10 years to recover. I was the saddest at that time. I couldn’t eat, I couldn’t sleep. It’s really painful. I dare not say anything, there was no money, I was very worried, thinner than now*.”

Financial security emerged as the second largest sub-theme within this domain. Participants discussed financial security and satisfaction as a key driver of and contributor to their well-being. The following quote showcases the importance placed on financial security by some participants in terms of their overall well-being.

“*Money is very important. Let me tell you, marriage and love life are very important, but money is more important. With enough money, there would be nothing to worry. Once you had the house, you don’t need to make a lot of money. Only to make enough to spend.*” Female participant

Housing-related issues constituted the third largest sub-theme within finances. Participants shared about the importance of owning a home, the stressors associated with having landlords that often-raised rent and/or did not keep up properties, the importance of helping family members and especially their children buy a home, and other aspects of home ownership as an important area of well-being.

##### Work-life

The third largest domain was work-life, representing 10% of total well-being mentions, and mentioned by 79% of participants. This domain included job-related tasks and their impact on health (e.g., inability to sleep, having little time for pleasurable activities or family time), and social relations from work as potential avenues for support and motivators for healthy behaviors. For instance, female participants often mentioned walking with other female co-workers or finding out about exercise classes and opportunities through co-workers. Participants also spoke about job-related stressors (e.g., conflicts with superiors, or pressures of having their own business) and about their jobs and/or careers as an aspect of purpose and meaning in their lives. Purpose and meaning derived from work was discussed across various occupations, from physicians to cleaning staff in our sample. The following quote highlights job related stress and negative impacts on well-being:

“*Because I am in public relations, a lot of activities. So, I have to stay up late, work overtime. It made me very tired, and at that time, it also made me very tired in my mind. My health started to have some problems. It was that time! It was tremendous. And the point was that, our boss would swear. It was not minor yelling. He used to swear. So, the pressure was tremendous*.” Female participant

##### Other well-being domains

The fourth most frequently discussed domain for well-being was lifestyle behaviors (9% of data elements), followed by sense of self (8% of well-being’s data elements), physical health (5% of well-being’s data elements), resilience (4% of well-being’s data elements) and religion and spirituality (2% of data elements). See Fig. 2 for further details regarding the percent of participants mentioning each domain.

#### Health findings

Figure 1, panel b, shows how participants discussed the concept of health. Physical health was discussed the most, accounting for 41% of the data elements from the health portion of the interview and discussed by 100% of participants. Disease-related comments were the most frequently mentioned sub-category within the physical health domain (28% of all data elements under physical health). Participants discussed a variety of issues including chronic health conditions, their related risk factors (e.g., high blood pressure, high cholesterol) and their impact on daily functioning; disease management; physical injuries; and other diseases, including issues such as colds or allergies. For instance, when asked to describe a time of poor health, a participant shared the following:

“*The worst was that I had diabetes! When I was hospitalized because of diabetes for insulin, that was the worst. I felt very depressed at first, and I let go. Anyway, I eat, take medication, only that I don’t exercise. I go to see the doctor regularly, do blood test, pick up the medication. Sometimes I don’t have breakfast, I only eat lunch. I used to watch my glycated hemoglobin count. I used to check daily, not anymore. When I found out that I had diabetes, I checked every day for a year. I stopped. Anyway, whatever the sickness, after a long time, you would get used to it. Now it’s mainly to have it well-controlled, just like this!*” Male participant

Health care use was also frequently discussed in the context of physical health. Participants spoke about use of both western medicine and traditional Chinese medicine approaches to managing diseases. Notably, they spoke more often about Western medicine (35 data elements) compared to traditional Chinese medicine (8 data elements), but generally spoke favorably about both. The following quote highlights a participant’s interactions with both health care systems:

“*One day, I decided to go to see the Chinese medicine doctor. Took the Chinese medicine for a week. Now, so many people have seen me and say that my hands are really all better*.” Female participant speaking about skin concern on her hands.

“*The medication is helpful, 2 tablets a day and it is under control*.” Male participant discussing diabetes management with Western medicine.

Aging-related changes in physical health were often brought up by participants (see Fig. 1, panel b). This included age-related changes such as menopause for our female participants; increased disease and comorbidities; weight gains over time; decreased ability to recover quickly after an injury or illness; increased number of aches; lower mobility or physical stamina; and changes in appearance.

“*I worked in Taichung, it was 20, 30, or 40 years ago. The former boss asked me to have dinner together with other co-workers. Everyone’s health is a lot worse now, with silver hair. We all are either grandmothers or grandfathers. We couldn’t recognize each other when we first met. I was so saddened, asked myself not to think too much. I have to let it go*.” Female participant

Within the physical health domain, participants also discussed issues related to the link between mental and physical health, vitality, pain, health literacy, social comparisons, and good physical health as a stepping stone to being able to engage in valued activities:

“*This is very important. You can't do anything, if you are not healthy, right? So, health is more important than wealth. I pay a lot of attention to be healthy. If you are not healthy, you can't be volunteers. And you can't do other things. So, we have to take care of ourselves to take care of others*.” Female participant discussing overall physical health during the health portion of the interview.

##### Lifestyle behaviors

The second most frequently discussed domain within the health portion of the interview was lifestyle behaviors, which accounted for 22% of the data elements and was mentioned by 88% of our participants. This domain included multiple health behaviors and daily practices such as physical activity, diet, self-care and leisure behaviors, and sleep. Participants discussed both engagement or lack of engagement in these behaviors and their influences on their health. The following quotes showcase a couple of these comments:

“*I am so afraid of being old, I do exercises. Go take a look at the secretary. She goes to the sports center. I asked the secretary how could you be so beautiful always? The secretary replied that she goes to exercise often, so I go to exercise too. I had my exercises already. I danced on Mondays, Wednesdays, and Fridays. Although I am not a very advanced dancer, it is always better than sitting on the couch and watching TV at home. At least three days a week, I go to exercise. If not, I will walk to the park or the playground for two laps or go sit and chat with everyone*.” Female participant highlighting positive social influences on her exercise.

“*I have this concept that the health is not based on western medicine, it comes from your normal three meals. In fact, my concept is that a normal diet for cancer patients is also the best chemotherapy. Because, the cells are in need of nutrients. I feel that everyone should take care of oneself. It is from the diet of three meals, should not drink. Don’t get those unhealthy drinks, including those delicate processed foods, don't take those*.” Male participant discussing diet.

##### Family

The third most frequently discussed domain within the health portion of the interview was family (11% of data elements in this section and mentioned by 79% of participants). Similar sub-themes were found in this section compared to the well-being portion of the interview. Participants highlighted their families as a key motivator for maintaining good health. A participant recalled the following after dealing with some health problems:

“*I lost weight. I didn’t dare to go out, and my health was very poor. I couldn’t cook, couldn’t do anything, I remember during that time, my mother and two sisters came to take care of me for a month. In less than a year, I could live by myself. I want to say that I couldn't fall apart. If I fell apart, my mother would have to come to take care of me. I can't let my elderly mother to take care of me for rest of my life. My children were still so young. So, I told myself that I had to get better soon*.” Female participant

##### Other health domains

As seen in Fig. 1, participants also mentioned, in decreasing order of frequency, work-life, resilience, finances, sense of self, and spirituality and religion during the health portion of the interview. See Fig. 2 for more details regarding % mentions and % of participants mentioning each.

### Post-coding analyses

#### Connections among petals

**Fig. 3:**
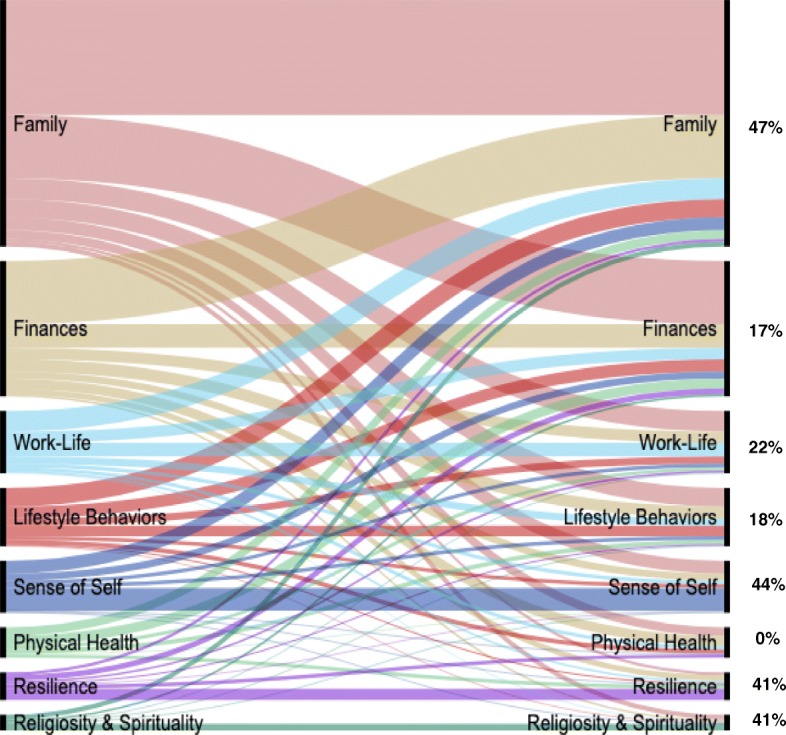
Connections among **well-being** domains *Legend*. Percentages on the right indicate the proportion of data elements in each domain that were single coded (not double-coded with another domain)

**Fig. 4:**
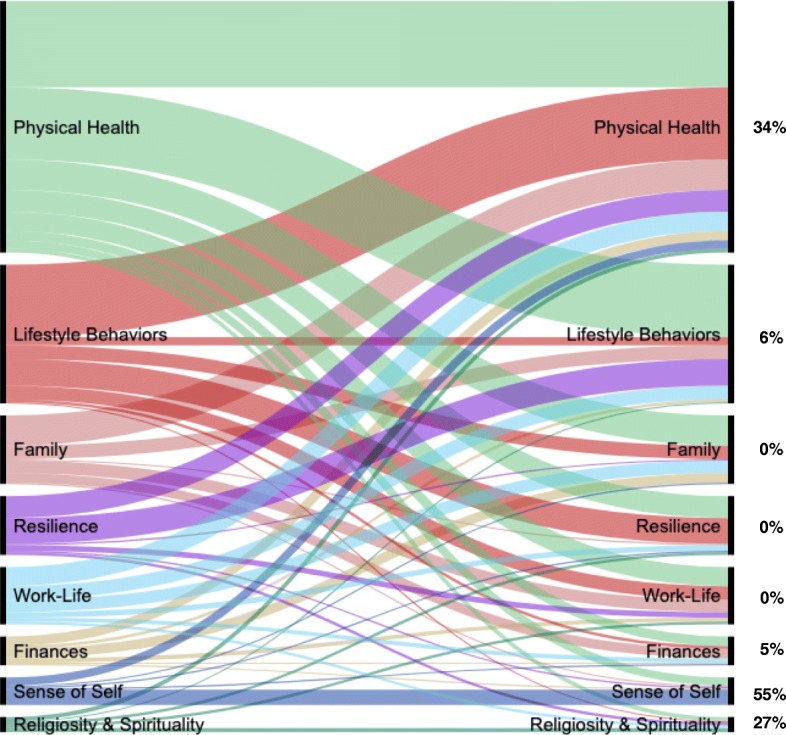
Connections among **health** domains *Legend*. Percentages on the right indicate the proportion of data elements in each domain that were single coded (not double-coded with another domain)

Although domains are presented in Fig. 1 as distinct petals for clarity purposes, participants often discussed multiple domains concurrently. Post-coding analyses revealed a pattern of complex interconnections shown in the form of Sankey diagrams [39, 40] in Figs. 3 and 4 for well-being and health, respectively. Straight lines connecting the same domains across the diagram indicate data elements that were only coded within that domain (i.e., no other domain discussed within the data element). The percentages shown in the figures (and also evident by the width of the lines) represent the proportion of data elements in that domain that were not double-coded. Curved lines connecting different domains across the graph indicate instances in which participants spoke about the two domains concurrently (i.e., double coding). Again, the thickness of these curved lines indicates the frequency of interconnectedness. As the figures show, mentioning multiple domains in the same data element was common. For example, for well-being, family was mentioned along with another domain 53% of the time. In the case of health, physical health was mentioned alongside another domain 66% of the time.

#### Contributors to and detractors from well-being and health

**Fig. 5:**
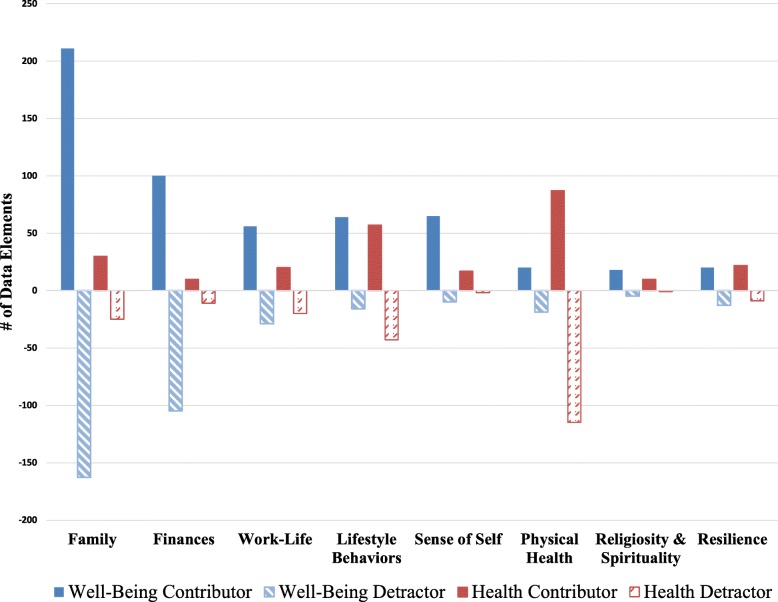
Understanding how each domain contributes to or detracts from well-being and health

Our post-coding analyses also explored how participants discussed each domain in terms of contributing to or detracting from their health and well-being. These findings are presented in Fig. 5. For instance, while family was most often discussed by individuals in terms of contributions to well-being (211 data elements), it was also discussed as a detractor (163 data elements). Many of these mentions of family as a detractor can be explained by our stress sub-theme, which captured pressures of raising young children, conflicts with in-laws, worries and issues regarding illnesses of family members, and stressors related to caring for elderly loved ones. Finances was also a domain discussed in terms of both contributions to and detractions from health and well-being. Some participants discussed financial stability as positively impacting their well-being and constituting “content” in their lives. Other times they spoke about financial concerns such as housing issues as detracting from their well-being. In the context of the health portion of our interview, physical health was more often discussed as a detractor than as a contributor (115 data elements vs 87).

#### Exploring low-ladder ratings on well-being and health

We further explored the interviews of participants who self-rated their well-being and/or health as being low or poor (i.e., ladder ratings ≤4 out of 8) in order to find information that could be useful in the development of targeted intervention for this sub-group. Seven participants rated their well-being and/or health within this range (*n* = 2 for well-being, *n* = 6 for health, and one with low ratings on both). In terms of demographics, males were overrepresented in this sub-sample (6 of 7 participants or 86%) compared to their proportion in the overall sample (46%). The one female on this sub-sample had a low rating on health, not well-being. While we found no overall differences in the frequency of mentions of domains for these participants, we did find differences in the content of two domains in the health portion of these interviews: physical health and lifestyle behaviors. Within the physical health domain, these participants were more likely to discuss the impacts of age-related changes (6 of 7 participants) compared to the overall sample (14 of 24 participants). They often tied their low health ratings to aging-related physical health symptoms including the development of chronic conditions such as diabetes, joint pain, and less energy and vitality. For lifestyle behaviors, all of these participants discussed lacking in exercise as being related to their current physical health issues. Many of them recalled involvement in sports or higher levels of physical activity at a younger age. However, they expressed current levels of inactivity due to work-related stress, lack of time and energy or other barriers. They also discussed difficulties with sleep quality as contributing to lower ladder ratings. Given the small sample of participants in this follow-up analysis, we encourage future studies exploring this information.

## Discussion

Using a grounded approach that draws from narrative inquiry, the present study explored the concepts of health and well-being among a sample of adults in Taiwan. Eight domains emerged for both constructs; however, participants spoke with differing levels of frequency about each of the domains. Family and finances were spoken about most often in the context of well-being, while physical health and lifestyle behaviors were discussed with more frequency when participants discussed health. Post-coding analyses revealed a pattern of connections among domains and ways in which domains both contribute and detract from health and well-being. Our findings offer insights into what it means to “be well” and have key implications for the science and measurement of well-being, and public health programs.

Our results highlight potential key differences between health and well-being. As seen in our flowers, domains were discussed with different frequencies when talking about health vs well-being. It is possible that increased frequency of discussion is an indication that some domains are more important or salient than others. Moreover, only a third of our participants rated their current health and well-being using the same ladder rung (see Table 1). This indicates that most participants clearly interpreted the terms to mean different things. Additionally, while participants spoke about disease-related issues in both the health and well-being portions of the interview (although to varying extents), they only spoke about interactions with health care systems in the discussions about health.

The frequency and content of the family domain are consistent with the importance of collectivistic values in Asian cultures, and in particular with the relevance of filial piety. A core concept in Confucianism, filial piety is characterized by conceptualizations of the family as an inseparable entity with complex roles, duties, respect, loyalty, and resource sharing among members [41, 42]. Studies have found specific filial behaviors to be predictive of well-being. For example, after controlling for health and financial strain, respect for parents was predictive of increased well-being among older adults in Hong Kong [41]. A similar construct, *familismo*, exists within the Latinx culture, representing the importance of family structures, pride, support and identity surrounding family values [43, 44]. Familismo has been found to be associated with both well-being and physical health [45]. Closeness and social support have received support as potential mediators in these associations [46]. This showcases a potential commonality or extension beyond Asian cultures.

In terms of financial issues and its relation to well-being, it is important to consider the various changes that have occurred in Taiwan over the last half century. The country has experienced rapid urbanization and industrialization, including a heavy emphasis on manufacturing and private enterprises, which marked a change from a prior reliance on agriculture [47, 48]. Other changes included women entering the labor force at high rates, large scale infrastructure projects to accelerate economic and social development in the 1970s, and the implementation of universal health care in the 1990s [21, 48]. Financial security emerged as a key well-being domain from our data, with participants often discussing finances as the second topmost concern after family. Housing stability emerged as a key sub-domain within finances. More data is needed to better understand how rapid changes in the country have influenced the relationship between financial stability and well-being, or whether other cultural values and context of individuals play a larger role in this relation. Nonetheless, our findings suggest that any efforts to ameliorate financial instability and improve housing can potentially have a large impact on the well-being of Taiwanese individuals. This is consistent with the Organisation for Economic Co-operation and Development (OECD) framework for well-being research, which highlights the importance of income and wealth, earnings and housing [49].

### Comparison with existing measures

Currently, there is little consensus regarding exemplary measures of health and wellbeing, and reviews suggest that much more work needs to be done in this area [50]. Our findings offer an opportunity for future refinement and rethinking of existing tools. For example, several popular measures of well-being (see e.g., the WHO Well-being Index-5 [51]) and the 12-Item Well-being Questionnaire [52] concentrate primarily on positive and negative affect or mood. While our participants did speak about affect, it was often in the context of discussing another domain (e.g., family, finances) and hence the experience of affect did not emerge as a separate domain from our data. These mentions were also not frequent enough to grant them a petal in our flowers when representing sub-domains within family or finances. Existing measures have also included other psychological aspects of well-being including positive social relations and purpose and meaning [53–55], and personal growth and self-acceptance [54]. Our findings support the importance of purpose and meaning in different areas of participants’ lives and the key role of social relations (e.g., family members and co-workers). Indeed, studies in Taiwan have indicated that social participation is globally beneficial to the psychological health and well-being of older adults [56, 57]. Our sense of self domain also aligns with the inclusion of areas similar to those in Ryff’s instrument [54].

Our findings support a multi-domain approach to health and well-being. Our participants spoke about various aspects of physical health and functioning, which are often emphasized in measurement tools, while also discussing other key areas of their lives (e.g., family, finances, work-life issues, resilience) as being important. The arrangement of domains is highlighted by our flowers. For instance, our well-being flower includes physical health, although to a smaller extent than our health flower, and our health flower includes non-physical health domains (e.g., family, work-life, finances), although to a smaller extent than our well-being flower. This multi-domain or multi-faceted approach has been embraced by some measures that have opted for a combination of physical and psychological concepts when measuring health or well-being [58], while others have added aspects of the cultural and social context under which individuals function in their everyday lives [59].

### Implications

A deeper understanding of what health and well-being means to individuals is key for guiding effective public health policies and health promotion efforts. As our flowers suggest, these concepts are quite expansive and include multiple domains in addition to physical health, disease-related issues or interactions with the health care system. Beyond population or aggregate level “tracking” of comprehensive health and well-being indicators that do not rely solely on experienced deficits, an important opportunity lies in grounding public health efforts and policies in the local population’s lived experiences and what really matters to people [17].

Health and well-being domains and their nuances can be translated into actionable steps and policies to promote optimal conditions for individuals to “thrive” [60]. Our findings point to the importance of efforts that promote family cohesion, economic and educational opportunities in order to promote financial stability, strengthening housing policies to make home ownership a potential reality while also protecting renters’ rights, and improving working conditions and programs that can promote social cohesion and engagement in healthy behaviors at work. Thus, while the domains identified in this study are person-centered (i.e., reflecting the personal experiences of a participant), the stories that participants offered provided insights into how well-being and health are influenced by structural factors. For instance, within the finances domain, participants spoke about housing-related issues which may depend on macro-level factors such as housing availability and income. These links to macro-level or structural factors can then be targeted to improve individuals’ health and well-being.

Our findings also point to the opportunity of using families as a key leverage point for effective health and well-being promotion. Programs that involve family members or frame engagement in healthier behavior as being for the overall well-being of the family can potentially be more effective in changing individual behavior. Our participants often spoke about family as a key motivator for living healthier lives and for improving or maintaining physical health. Thus, programs and interventions that consider the role of families in building individuals’ resilience would likely be of greater effectiveness than programs that do not incorporate the family. For instance, a family-based nutrition health education program for hypertensive patients in Taiwan was found to be effective in lowering blood pressure, decreasing weight, and significantly reducing stroke-related risk factors [61]. As part of this program, all family members were educated on tips for modifying unfavorable lifestyle behaviors (e.g., nutrition), which resulted in a significant stroke risk reduction compare to the control group that did not incorporate the family. Additionally, these programs could be cost-effective as they impact various members of the family (not just the focal individual) and help shape the behaviors of children and future generations [62, 63].

Similarly, employment settings offer another leverage point to influence health and well-being and prevent future disease burden [64, 65]. For instance, our participants discussed using employment facilities for exercise, being supported by others at work to engage in various healthy behaviors, having positive social relations and engaging in self-care or other enriching activities such as religious services. This supports the need for workplace activities that can enhance well-being such as walking groups, sharing healthy recipes, access to recreational or exercise facilities, access to religious and spiritual services, and others.

Our findings also suggest that health care services, as they currently exist, are not fully addressing important aspects of health and well-being. Most health services continue to organize their efforts around disease management and/or prevention and the presence of deficits. Other aspects of well-being are either much less integrated or perhaps non-existent, for example religious/spiritual counseling or support, or programs for enhancing purpose and meaning or financial assistance. If the goal is to optimize health and well-being, more comprehensive services and programs that allow for the incorporation of both assets and deficits are needed. While still in the beginning stages, some health care systems are indeed adapting more integrated strategies that consider more holistic approaches to the creation and maintenance of health and well-being [66].

### Limitations

The present study has several limitations, including the small convenience sample, which limits the generalizability of the findings. Thus, it is important to interpret the findings of the current study as exploratory and hypothesis generating. Also, our interviews were structured to first ask participants about well-being. It is possible that during the discussion regarding health, participants were experiencing fatigue or had fewer things to discuss given that they had already shared a substantial amount of information. The total number of data elements for each section is evidence of much shorter discussions during the health portion of the interview. Lastly, translation of abstract, complex concepts such as well-being and health can be difficult. It is possible that a different translation of “well-being” and “health” would have led to different results. Thus, we caution cross-cultural well-being researchers to carefully consider their translations and to be transparent about their chosen terms in any dissemination efforts. Moreover, our interview transcripts were translated into English for coding, potentially losing some richness or context in this effort. Nonetheless, we believe this exploratory study offered rich data regarding participants’ lived experiences with health and well-being and highlights key areas for additional exploration.

### Future directions

Future studies should consider using our methods to explore health and well-being among other Asian countries and/or with other demographic groups in order to increase generalizability and explore potential sub-group differences. This is particularly important before using findings to inform public health programs or policies. Future studies should investigate in more depth key constructs highlighted by our study (e.g., various aspects of family and their role well-being, or the role of diseases and ageing-related changes in the experience of physical health) with different methodologies to better be able to test more specific hypotheses and examine the impacts on key relevant public health outcomes. Moreover, comparisons among groups with different socio-demographic characteristics is important. Prior studies have found, for example, that filial piety is more highly endorsed in Taiwan and Hong Kong among individuals with lower socioeconomic status [26]. Finally, we encourage additional studies that follow-up on our exploratory findings related to participants who rated themselves as low on health and/or well-being in order to develop targeted interventions for this sub-group.

Additionally, some evidence points to the importance of future research that can disentangle the relative contributions of different sources of well-being. For instance, studies have highlighted potential differences between individual and relationship-based influences on health and well-being [24]. Our findings supported a mixture of individualistic and collectivistic sources of well-being with family in particular being a predominant relationship-based influence. In this sense, our participants often derived well-being from their interconnections to others, but also discussed freedom of choice and independence as contributors to their well-being.

Furthermore, as modernization and Western influences continue to permeate Asian cultures, there is a key opportunity for prospective studies to explore the impacts of these changes on the health and well-being of different populations. For example, what impact do changes in family structures, such as the movement toward nuclear families seen in Taiwan and other East Asian counties [41], have on health and well-being? Lastly, cohort effects and differences based on demographics (e.g., gender, income, education) should be further explored. For example, given the societal changes highlighted here, it is possible that younger generations would endorse traditional values, such as filial piety, to a lesser degree, leading to differences in what domains are more salient to them.

## Conclusions

Optimizing health and well-being is a valued and increasingly important goal. A better understanding of what it means to be well and to live a fulfilling life can offer insights into specific leverage points to inform more effective policies, practices and interventions. The present study highlights the importance of grounding understanding of health and well-being in the cultural and social context of the individuals we are trying to serve.

## Data Availability

The data used during the current study, and original and translated versions of the interview guide, are available from the corresponding author on reasonable request.

## Abbreviations

FJU: Fu-Jen Catholic University
OECD: Organisation for Economic Co-operation and Development
WELL: Wellness Living Laboratory
WHO: World Health Organization
